# A Unique Presentation of Acute Biliary Ascites Due to Spontaneous Biliary Duct Perforation With Bowel Obstruction: A Case Report

**DOI:** 10.7759/cureus.61700

**Published:** 2024-06-04

**Authors:** Vivek R Velagala, Aayushi Bhatnagar, Jayant D Vagha, Sham Lohiya, Ajinkya Wazurkar, Shailesh Wandile, Chaitanya Kumar Javvaji

**Affiliations:** 1 Medicine, Jawaharlal Nehru Medical College, Datta Meghe Institute of Higher Education and Research, Wardha, IND; 2 Pediatrics, Jawaharlal Nehru Medical College, Datta Meghe Institute of Higher Education and Research, Wardha, IND

**Keywords:** roux-en-y hepaticojejunostomy, subcapsular abscess, partial situs inversus, abdominal distention, peritoneal irritation, abdominal paracentesis, bowel obstruction, biliary ascites, biliary peritonitis, spontaneous biliary duct perforation

## Abstract

Biliary ascites due to spontaneous biliary duct perforation is a rare case presentation usually seen in the paediatric age group of 6-36 months. We are presenting the case of a 14-month-old baby with abdominal distention associated with abdominal pain, vomiting, fever, and a history of no passage of stools. Upon examination, the abdomen was tense and tender. On radiological investigations, gross free fluid was present in the abdominal cavity along with bowel obstruction and partial situs inversus of the spleen and stomach. The bowel obstruction was relieved by rectal stimulation, after which oral feeds were well tolerated. Bilious fluid was found on diagnostic paracentesis, confirming the diagnosis. The patient was managed further by broad-spectrum antibiotics and drainage of the free fluid. The management ranges from conservative treatment to Roux-en-Y anastomosis. A non-surgical diagnosis is uncommonly seen and helps improve the patient's prognosis if detected early. This case report highlights the importance of early diagnosis and non-surgical treatment modality in critical patients.

## Introduction

Biliary ascites is a rare condition that involves the accumulation of biliary ascitic fluid in the abdominal cavity and can cause peritoneal irritation, leading to secondary infection. Biliary ascites is one of the less commonly observed types of ascites. The aetiology is either due to trauma or due to congenital malformation, apart from spontaneous biliary duct perforation (SBDP). The perforation may often not be visible in radiological investigations, which makes the diagnosis difficult. SDBP is primarily seen in age groups from six months to 36 months. SBDP has a male-to-female ratio of 1.2:1 [1]. SBDP in the paediatric age group has an ambiguous aetiology. Most reported cases are attributed to congenital malformation of the biliary ducts or congenital weakness [1]. The malformation results in an unusual mixing of the bile and pancreatic juices, forming a protein plug, hence blocking the ducts, which manifests as abdominal pain [2]. The spectrum of symptoms includes abdominal distention, jaundice, nausea, vomiting, ascites, and peritonitis [3]. Hepatobiliary technetium-99m-iminodiacetic acid scintiscan is the investigation of choice in diagnosing biliary leak, but its availability is the limiting factor in third-world countries [4]. The diagnosis can be differentiated based on contrast-enhanced computed tomography (CECT) but requires confirmatory diagnostic paracentesis [5]. The management includes non-operative and surgical techniques. The non-operative measures include broad-spectrum antibiotics and drainage. The surgical procedure includes Roux-en-Y anastomosis, which is the most commonly used option of management [2].

## Case presentation

A 14-month-old female baby came to our tertiary healthcare centre's paediatrics department in central India with complaints of abdominal distention and multiple episodes of vomiting for five days. She had not passed stools and had a high-grade fever for four days. The baby is third by birth order, born out of a non-consanguineous marriage. As narrated by the mother, the child was asymptomatic five days back when she started having abdominal distention, insidious in onset and gradually progressive in nature. Initially, the child had multiple episodes of vomiting containing food particles, non-bilious in nature, and non-blood tinged. The child was unable to tolerate any oral feeds. After one day, the child started developing a documented high-grade fever, which was insidious in onset, associated with chills, and relieved by taking medication with no diurnal variation. The child also had not passed stools for the past four days. For the past two days, the child started having multiple episodes of vomiting, which was foul-smelling. With these complaints, the child was brought to our hospital.

After obtaining proper consent for examination after admission, the child had tachycardia with a heart rate of 150/min and tachypnoea with a respiratory rate of 40/min with no signs of shock. On examination, gross abdominal distention with tenderness was present. Bowel sounds were audible. No signs of peritoneal irritation were seen. On rectal stimulation, the child passed stools. Blood investigations showed a low haemoglobin (7.7 gm%), a high total leukocyte count (TLC) (32,700/cmm), a high platelet count (5 lakhs/cmm), and a high C-reactive protein (193 mg/L). Liver enzymes and renal function tests were within standard limits. Serum amylase and lipase were also normal. Dengue NS1 antigen was negative. The child was kept nil by mouth and started on IV dextrose normal saline, injection meropenem (40 mg/kg/day), and injection vancomycin (60 mg/kg/day). Paracetamol (15 mg/kg/dose) was also started. On inserting Ryle's tube, the child had black-coloured altered aspirate. The quantity of aspirate was almost 100-200 ml in the initial few days, which later decreased.

**Figure 1:**
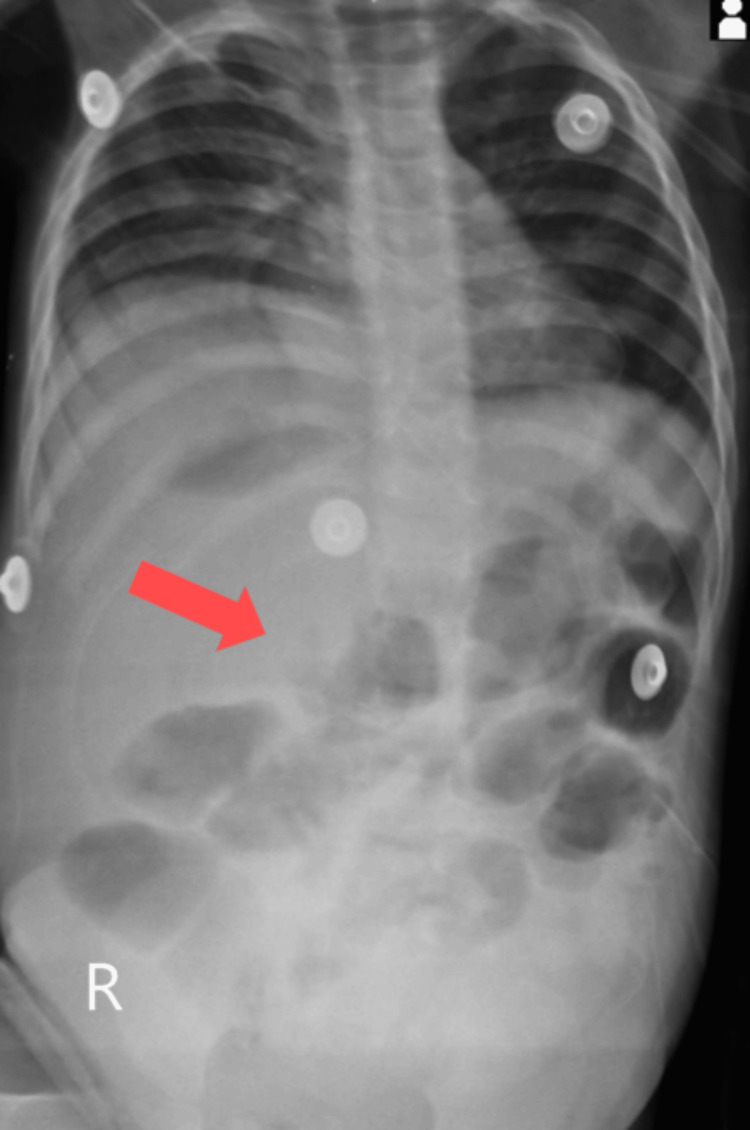
Abdominal X-ray showing distended bowel loops indicative of obstruction (red arrow).

**Figure 2:**
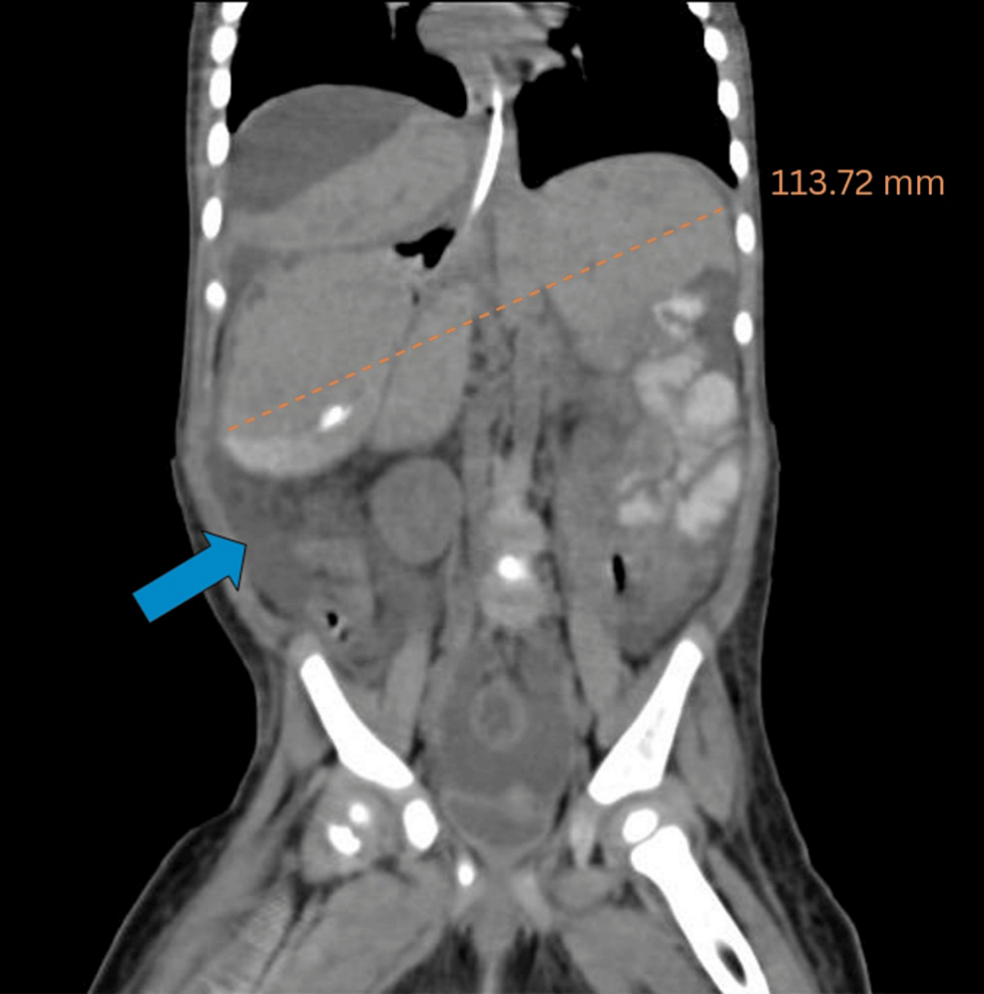
Coronal view of abdominal CECT showing hepatomegaly (orange dotted line) and ascites (blue arrow). CECT: contrast-enhanced computed tomography

**Figure 3:**
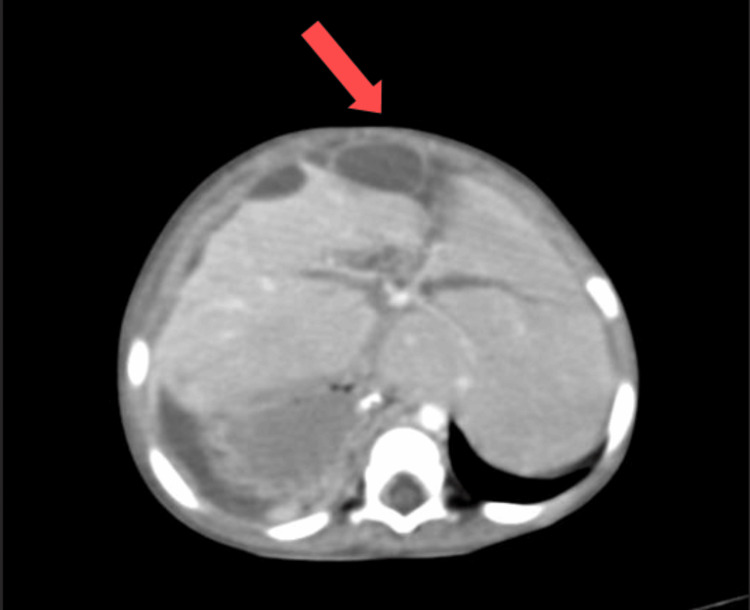
Axial view of abdominal CECT showing subcapsular hypodense collections (red arrow). CECT: contrast-enhanced computed tomography

Abdominal X-ray showed gross dilation of bowel loops (Figure 1). CECT of the abdomen suggested gross ascites with a distended abdomen with the possibility of secondary obstruction, and a moderate hepatomegaly of 113.72 mm was noted (Figure 2). Multiple subcapsular hypodense collections causing scalloping of the liver surface were also observed (Figure 3). CECT did not show a prominent common bile duct and intrahepatic biliary radicles. Partial situs inversus was noted.

Pigtail catheter insertion was done under ultrasonography (USG) guidance in the subdiaphragmatic and pelvic abscess. There was a drainage of bilious green-coloured fluid. Analysis showed a total leukocyte count of approximately 11200/cmm with a predominance of neutrophils. Ascitic fluid biochemical analysis showed lactate dehydrogenase (LDH) of 607 IU/L, fluid protein of 2.3 g/dL, and bile salts present. Blood culture and ascitic fluid culture suggest no growth of any organism. Therapeutic paracentesis guided via USG was done to remove the fluid collection. The symptoms of obstruction resolved gradually when the child passed stools on rectal stimulation. As the symptoms decreased, feeding by mouth was well tolerated, and the patient was subsequently discharged after two weeks in the hospital. The patient has been asked for a follow-up after three months.

## Discussion

A review of the existing literature shows that spontaneous perforation is rare, with the highest incidence in the infantile age group, extending to 36 months of age. There is a known association between Ivemark syndrome, Hodgkin lymphoma, necrotising enterocolitis, acquired biliary atresia, and choledochal cyst [1]. Less than 100 cases have been reported worldwide [3]. No existing literature exists on the association between biliary perforation and partial situs inversus, making this presentation unique. The path to the diagnosis of spontaneous biliary peritonitis is rarely triggered by clinical suspicion alone, requiring the clinician to perform a CECT scan of the abdomen. The gold standard diagnostic investigation is hepatobiliary technetium-99m-iminodiacetic acid scintiscan, whose availability is a limiting factor in the immediate diagnosis, especially in third-world countries. According to the current literature, a paediatric patient with known biliary tract abnormalities or free fluid in the abdomen, either localised or generalised on radiological imaging, along with jaundice without pneumoperitoneum, suggests biliary perforation without the need to perform scintiscan [4,5]. The perforation size varies from a punched-out appearance to a size not visualised in radiological investigations [3]. In this patient, the perforation was not visible in radiological investigations, but as per the current literature, it does not exclude the diagnosis. Paediatric SBDP is especially difficult to diagnose early as the bile that leaks into the abdomen is sterile and hence leads to inconspicuous peritoneal irritation, as seen in this patient. The peritoneal irritation with fever and other symptoms of secondary infection sets in at a later stage [2]. Jaundice can be a vital symptom generally observed in patients who have an intrahepatic biliary perforation. Still, few studies show that if the perforation relieves the cholestasis, a typical obstructive jaundice will not be seen [2,6].

In this patient, the clinical suspicion was brought forth due to the spectrum of symptoms of vomiting and abdominal distention coupled with laboratory findings such as raised bilirubin in ascitic fluid, high leukocyte, high C-reactive protein, and mildly elevated serum alanine aminotransferase (ALT) and aspartate aminotransferase (AST). The findings in this patient coincide with existing literature for a differential diagnosis [3]. We conducted radiological investigations to assess this unique presentation that showed free fluid. The diagnosis was confirmed after paracentesis, with bile as aspirate. The presence of dilated bowel loops in the X-ray suggested obstruction, underscored by the history of no passage of stools and increased risk of a subsequent umbilical or inguinal hernia, making the diagnosis and immediate intervention imperative [5]. There was hepatomegaly noted with a measurement of 113.72 mm. The normal range falls between 63 mm (third percentile) and 111 mm (97th percentile) [7]. The differential diagnosis includes a sizeable choledochal cyst and perforation of the gallbladder, which were both ruled out on subsequent CECT showing the presence of free fluid [8]. The free fluid could also indicate a differential diagnosis of ascites due to any hepatic cause but was ruled out based on biochemical and paracentesis results [3]. Spontaneous biliary perforation is one of the causes of surgical jaundice in children. From the literature reviewed, the management ranges from conservative treatment using catheter drainage to cholecystectomy or Roux-en-Y hepaticojejunostomy. Surgical management is more commonly performed than conservative management [1]. This case was managed conservatively, using antibiotics, antipyretics, and drainage tubes to prevent further bile leak into the peritoneum. Although rarely possible, similar management has been followed in the literature studied [4]. Conservative management was an option only because of early presentation, early detection, early alleviation of bowel obstruction, and lack of congenital malformation [8].

## Conclusions

Diagnosing biliary ascites depends on clinical suspicion as the investigation of choice for bile leak, hepatobiliary technetium-99m-iminodiacetic acid scintiscan, is less accessible. The majority of the reported cases pertain to spontaneous perforation, yet the aetiology remains a mystery. The diagnosis can be made without a scintiscan using clinical suspicion, laboratory findings, and radiological investigations confirmed by diagnostic paracentesis. This case report highlights the unique presentation of biliary ascites with bowel obstruction along with the challenges faced for diagnosis. It throws light on the need for investigations for bile leaks that are more accessible for an early diagnosis and to prevent complications such as inguinal hernia, staining of the abdomen, shock, and necrosis.
